# Diffusion-weighted imaging reversal after reperfusion therapy in acute ischemic stroke: associations with white matter hyperintensities and cerebral microbleeds

**DOI:** 10.1186/s42466-026-00545-w

**Published:** 2026-10-29

**Authors:** Nora Bannert, Paula Klassen, Regina Krijatov, Amir Dooat, Anastasia Nosanova, Jens Dreyhaupt, Hans-Peter Müller, Karl Georg Haeusler, Mona Laible

**Affiliations:** 1 Department of Neurology, University Hospital Ulm, Oberer Eselsberg 45, Ulm, 89081 Germany; 2 Institute for Anatomy and Cell Biology, University of Ulm, Ulm, Germany; 3 Institute of Epidemiology and Medical Biometry, University of Ulm, Ulm, Germany; 4 Department of Nuclear Medicine, School of Medicine, University Hospital Ulm, Ulm, Germany; 5 Department of Diagnostic and Interventional Radiology, University Hospital Ulm, Ulm, Germany; 6 German Center for Neurodegenerative Diseases (DZNE), Ulm, Germany

**Keywords:** Stroke, Thrombolysis, Thrombectomy, DWI reversal, White matter hyperintensities, Cerebral microbleeds

## Abstract

**Background:**

White matter hyperintensities (WMH) and cerebral microbleeds (CMBs) are common MRI markers of chronic small-vessel brain injury and have been associated with adverse outcomes after ischemic stroke. However, whether these markers influence early ischemic tissue reversibility following reperfusion therapy remains unclear. We therefore investigated their associations with diffusion-weighted imaging (DWI) lesion reversal, hemorrhagic complications, and short-term functional outcome after MRI-guided reperfusion therapy.

**Methods:**

In this retrospective single-center study, consecutive patients with acute ischemic stroke undergoing MRI-guided intravenous thrombolysis, with or without additional mechanical thrombectomy, were included. According to the institutional protocol, follow-up MRI was planned 24 ± 6 h after treatment to assess DWI lesion evolution, WMH volume, and CMBs. DWI reversal was defined as a reduction of ≥ 25% in baseline DWI lesion volume. Multivariable regression analyses were performed to identify predictors of DWI reversal, intracerebral hemorrhage, and functional outcome at hospital discharge.

**Results:**

Among 345 patients (median age 74 years [IQR 63–82], 48.1% female), 45 (13%) additionally underwent mechanical thrombectomy. DWI reversal occurred in 76 patients (22.0%) and was not associated with baseline WMH volume, CMBs, initial infarct volume, treatment modality, or thrombolysis time window. Higher baseline NIHSS and modified Rankin Scale scores were associated with lower odds of DWI reversal, whereas hypercholesterolemia was independently associated with increased odds of DWI reversal. We found no evidence of an association of WMH burden or CMB presence with intracerebral hemorrhage or of an independent association with functional outcome. Fourteen patients (4.1%) developed new CMBs on follow-up MRI.

**Conclusions:**

We found no evidence of an association between baseline WMH volume or CMB presence and early DWI lesion reversal or hemorrhagic complications following reperfusion therapy in this cohort. These findings provide no evidence that these two chronic MRI markers are major determinants of early infarct tissue reversibility. Larger multicenter studies incorporating comprehensive small-vessel disease phenotyping and long-term follow-up are warranted.

**Supplementary Information:**

The online version contains supplementary material available at https://doi.org/10.1186/s42466-026-00545-w.

## Introduction

According to the World Health Organization (WHO), 87% of all strokes are ischemic in origin [1]. Systemic intravenous thrombolysis is an established and guideline-compliant component of acute stroke therapy, and its effectiveness has been proven in randomized controlled trials [2–5]. The majority of imaging-based acute stroke studies rely on computed tomography (CT) because of its widespread availability in the emergency setting, whereas diffusion-weighted magnetic resonance imaging (MRI) is considered the most sensitive imaging modality for detecting acute ischemic stroke and specifically changes of the white matter [6, 7].

The term “cerebral small vessel disease” (CSVD) refers to a group of different vascular diseases affecting the smallest cerebral vessels, caused by various pathomechanisms [8]. It shows increasing prevalence in the elderly, affecting approximately 5% of all people under the age of 50 and almost all people over the age of 90 years [9]. CSVD may manifest itself with clinical symptoms. The neurological and clinical manifestations of CSVD include strokes and cognitive impairment, ranging from reduced cognitive performance to vascular dementia [10, 11]. Physical disabilities such as gait disturbances and urinary incontinence [11, 12] as well as psychiatric disorders such as depression and behavioral disorders also occur [8]. Endovascular cerebral damage in the context of CSVD cannot be directly detected in either CT or MRI imaging. However, it is possible to analyze the presence and progression of CSVD on the basis of cerebral microangiopathies, which become noticeable histopathologically, but also in vivo by MRI features as cerebral microbleeds (CMB) and white matter hyperintensities (WMH) [11, 13].

Due to methodological limitations, the investigation of the impact of cerebral microangiopathies on the safety and efficacy of intravenous thrombolysis using alteplase is limited, as brain imaging was largely performed using initial CT scans and CSVD analysis was performed only occasionally, such as by Fazekas et al. [6]. Retrospective brain MRI findings were therefore used to evaluate the safety and efficacy of intravenous thrombolysis in the presence of CSVD [6, 11]. A current review and meta-analysis suggests that patients with the presence of at least one MRI-marker of CSVD are at an approximately 2.4-fold higher risk of developing symptomatic intracerebral hemorrhage (sICH) after thrombolysis, compared to patients with no evidence of CSVD [14]. Among the CSVD markers, CMBs have been most extensively reported in relation to sICH and stroke, while WMH were analyzed to a lesser extent [15]. Despite an increased risk of sICH and a higher probability of poor functional outcome, thrombolysis in the presence of CMB still seems to be related to an acceptable augmentation of risk for hemorrhagic complication. Therefore, the presence of a limited number of CMBs alone is generally not considered a contraindication to thrombolytic therapy, whereas current ESO recommendations advise against intravenous thrombolysis in patients with a high burden of CMBs (> 10) within 4.5 h of stroke onset [16]. Further findings from recent prospective randomized-controlled studies and a retrospective single-center study showed that it is not only possible to reduce the penumbra (defined as mismatch between diffusion- and perfusion weighted imaging) under thrombolytic therapy (with or without additional thrombectomy), but the prospectively collected international, multicenter, observational DEFUSE2 registry showed that in stroke patients with penumbra identified and saved, there was a favorable outcome [17, 18]. Evidence for cytotoxic edema in diffusion-weighted imaging (DWI) has appeared to be reversible [19], contradictory to previous pathophysiological theories. Interestingly, reversibility of DWI lesion volume has been found more frequently in ischemic stroke patients undergoing thrombolysis than in stroke patients without thrombolysis [18, 20–23].

Importantly, successful macrovascular recanalization does not necessarily result in complete tissue reperfusion, as persistent microvascular no-reflow and ischemia/reperfusion injury may limit tissue recovery despite restoration of flow in the occluded artery [24]. The aim of our single-center study was to use highly standardized serial MRI data from patients with acute ischemic stroke undergoing intravenous thrombolysis, with or without additional thrombectomy, to investigate DWI lesion reversibility. The cohort was not restricted to strokes attributed to small-vessel occlusion; rather, WMH and CMBs were assessed as chronic background MRI markers irrespective of the presumed etiology of the index stroke. We investigated whether WMH burden and CMB presence were associated with DWI reversal and, as secondary outcomes, hemorrhagic complications and short-term functional outcome after reperfusion therapy.

## Methods

### Study design

The retrospective, single-center study was conducted between 2011 and 2021 at the Department of Neurology at University Hospital Ulm, Ulm, Germany. The study was approved by the Ethics Committee of Ulm University (ethics application number 158/22). Due to the retrospective character of this study, consent to participate was waived by the Ethics Committee.

The final study cohort consisted of 345 patients with acute ischemic stroke. During the study period, MRI was part of the local standardized in-hospital imaging protocol for patients with suspected acute ischemic stroke and was not selectively requested at the individual neurologist’s discretion instead of CT. Inclusion criteria were age ≥ 18 years, an ICD-10 diagnosis of I63.X, intravenous thrombolysis (0.9 mg/kg bodyweight alteplase, 10% as bolus and 90% over 60 min) after admission MRI, and technically adequate follow-up MRI planned according to the institutional standard operating procedure 24 ± 6 h after treatment, with DWI evaluable for volumetry at both time points. Patients treated within 4.5 h of stroke onset were eligible according to the conventional time window; beyond 4.5 h, treatment selection was based on PWI-DWI or DWI-FLAIR mismatch according to prior studies [25, 26]. This imaging-based criterion was fulfilled by 101 patients (29.3%). Additional thrombectomy was performed following interdisciplinary clinical and neuroradiological assessment.

Brain images were evaluated for the presence of bleeding complications based on the Heidelberg Bleeding Classification [27]. sICH was defined as a neurological deterioration of ≥ 4 points on the National Institutes of Health Stroke Scale (NIHSS) score [28].

### Clinical characteristics

Patient data were collected from discharge letters, laboratory data, MRI images, and electronic patient records. Data included age, sex, time between MRI scans, stroke location and vascular territory, alteplase dose, premedication including pre-stroke statin use, cardiovascular risk factors, disability (modified Rankin Scale, mRS), and stroke severity (NIHSS). NIHSS was recorded at admission and at approximately 24 h, corresponding to the follow-up imaging interval, when available. Stroke etiology was assigned at hospital discharge after the diagnostic work-up according to the TOAST classification [29]. Thrombectomy data included recanalization results and onset-to-treatment times. Pre-stroke mRS was not systematically documented in the analytical dataset.

### MRI and evaluation

We analyzed the following MRI sequences at baseline and after thrombolysis: T2fl2D, a T2-weighted fluid-suppressed two-dimensional MRI sequence, T2TIRM, a T2-weighted turbo inversion recovery magnitude sequence, and DWI, the diffusion-weighted imaging sequence, which brightly displays the restricted movement of water in tissue. The images are taken in individual slices, i.e., two-dimensionally, which are later assembled into a three-dimensional image. In the first step, T2fl2D, T2TIRM, and DWI sequences were visually assessed in a PACS (Picture Archiving and Communication System, GE Healthcare, Chicago, Illinois, USA) to ensure the necessary image quality. Images were then stored as Digital Imaging and Communications in Medicine (DICOM) files for further use. The MRI images were evaluated using Tensor Imaging and Fiber Tracking (TIFT) software (extension to the in-house developed software, version 2025; H.P. Müller, University of Ulm, Germany) [30], which enabled MRI images consisting of so-called voxels to be transformed into a three-dimensional model. Brains were then aligned along the AC-PC line, an axis from the anterior commissure to the posterior commissure, to compensate for any possible tilting of the head during image acquisition.

The voxels were displayed in varying intensities to differentiate between WMH, microbleeds, the acute ischemic lesion, and normal brain tissue. This enabled marking and subsequent evaluation of voxels within the defined threshold range, including the DWI-hyperintense, ADC-hypointense acute ischemic lesion and WMH. Marking and determination were performed in three-dimensional space, allowing volumetric measurement. DWI and WMH volumes were measured in mm³. DWI reversal was defined a priori as a relative decrease of ≥ 25% in total DWI lesion volume between baseline and follow-up MRI. In patients with multiple acute DWI-positive lesions, all acute lesions were segmented and summed; reversal was therefore assessed on the basis of total lesion burden rather than an individual index lesion. Measurements were performed by trained raters (N.B., R.K., A.D., and M.L.). CMBs were defined according to Standards for Reporting Vascular Changes on Neuroimaging (STRIVE) [10] and assessed using the Brain Observer MicroBleed Scale (BOMBS) [31].

Inter-rater agreement for DWI lesion volume measurements was assessed between two independent raters in 30 patients. Bland-Altman analysis was performed on log-transformed volumes to obtain ratio-based limits of agreement. Reliability was calculated using the intraclass correlation coefficient (ICC; two-way random-effects model, absolute agreement, single measures) and Lin’s concordance correlation coefficient (CCC), both reported with 95% confidence intervals. ICC values were interpreted as excellent [32].

### Statistical evaluation

Continuous variables were expressed as median and interquartile range (IQR). Group comparisons were performed using the Mann-Whitney U/Wilcoxon rank-sum test and Wilcoxon signed-rank test for paired comparisons. Categorical variables were described using absolute frequencies and percentages and compared using the chi-square test or Fisher’s exact test as appropriate. The Kruskal-Wallis test was used to compare WMH volumes across numbers of cardiovascular risk factors, followed by Dunn’s test with Bonferroni correction. Binary logistic regression analyses were used to analyze predictors of binary outcomes. To assess the robustness of the predefined DWI reversal threshold, a sensitivity analysis used a more stringent ≥ 50% reduction in DWI lesion volume. A two-sided p-value < 0.05 was considered statistically significant. To examine potential confounding by vascular territory, the treatment–reversal association was additionally assessed using territory-stratified analyses, a Mantel–Haenszel estimate, and logistic regression including vascular territory. DWI reversal was also compared across TOAST etiological categories. Pre-stroke statin use was examined as a potential confounder of the association between hypercholesterolemia and DWI reversal.

Early neurological improvement (ENI) was assessed approximately 24 h after treatment, contemporaneously with follow-up MRI, and was defined as a decrease in NIHSS score of ≥ 4 points from baseline or an NIHSS score of 0–1 at 24 h. The association between DWI reversal and ENI was additionally assessed using multivariable binary logistic regression adjusting for baseline NIHSS and treatment modality (intravenous thrombolysis alone vs. intravenous thrombolysis plus mechanical thrombectomy).

Due to the exploratory nature of this study, all results from statistical tests have to be interpreted as hypothesis-generating and not as confirmatory. All statistical analyses were performed with SPSS, version 29.0.2.0 (IBM, Armonk, USA) and R, The R Project for Statistical Computing, version 4.6.0 by The R Foundation.

## Results

**Fig. 1:**
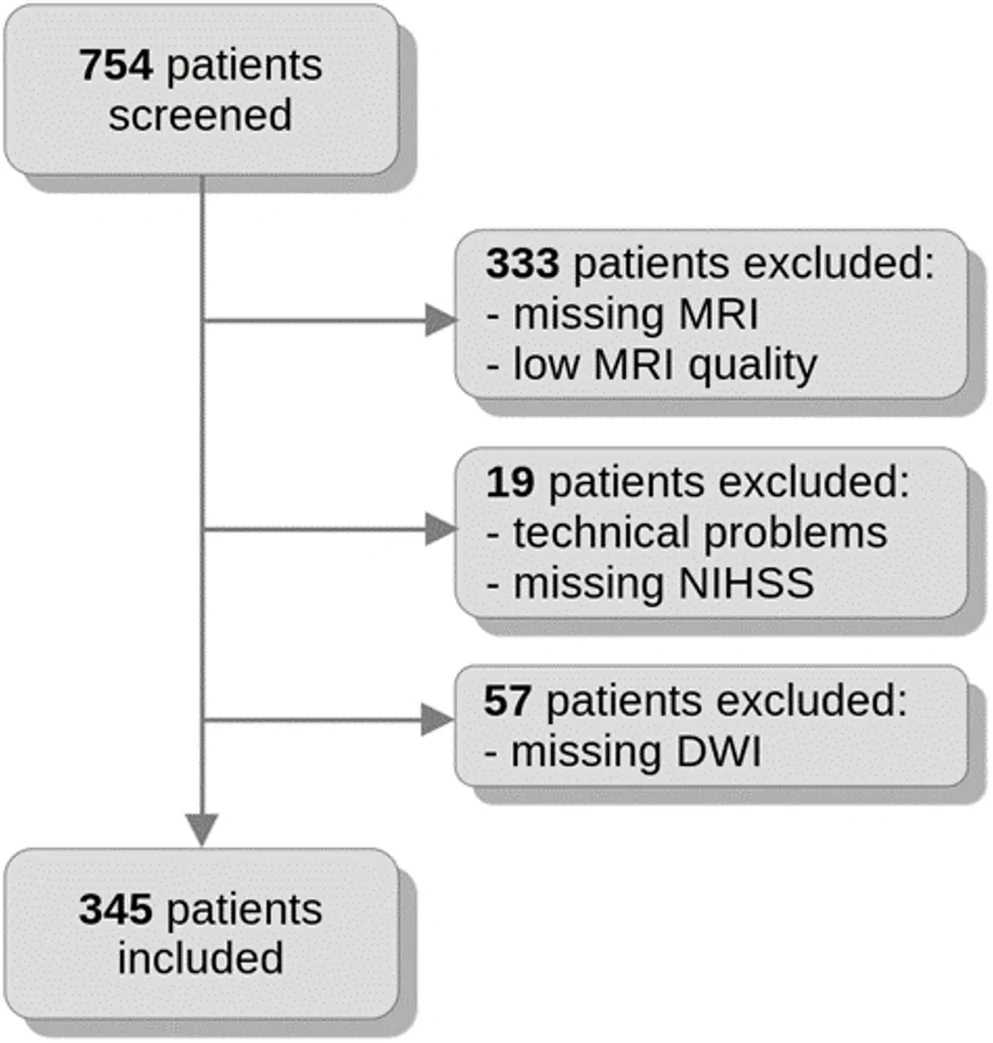
Study flowchart. Of 754 patients with acute ischemic stroke undergoing thrombolysis, 333 were excluded due to insufficient MRI image quality (44.2%) at least of one time point or substitution of MRI by CT imaging at least one time-point (*n* = 103); no MRI images in the acute setting (*n* = 188), as well as repetitive MRI imaging beyond the defined time frame (*n* = 42). In addition, 19 patients were excluded due to technical reasons (*n* = 5 due to failed data export from GE PACS, further *n* = 14 due to incomplete data (conversion). Of the remaining 402 patients, a further 57 patients had to be excluded from final analysis as a missing DWI lesion volume at baseline or follow-up prevented the calculation of DWI reversal status, resulting in a final study cohort of 345 patients with two available brain MRI scans, before and after thrombolysis

Of 754 screened patients with acute ischemic stroke undergoing thrombolysis, 345 met the inclusion criteria and had technically adequate baseline and follow-up MRI with DWI volumetry available at both time points (Fig. 1).

### Patient characteristics

Of 345 patients undergoing thrombolysis, 45 (13%) also underwent thrombectomy. Patient characteristics are described in Table 1. The median age of the entire patient cohort was 74 years (IQR 63–82), and 166 (48.1%) were female.

Time between the two MRI scans was in median 23.02 h (IQR 18.02–26.18).

Inter-rater agreement for DWI volume measurements was high. The ratio bias was 1.009 (95% CI 0.949–1.073), with ratio limits of agreement from 0.75 to 1.37. Reliability was excellent, with an ICC (2,1) of 0.993 (95% CI 0.984–0.997) and a CCC of 0.992 (95% CI 0.983–0.996).

**Table 1:** Patient characteristics based on treatment

	Total	Thrombolysis	Thrombolysis + thrombectomy	*p*-value
**N**	345	300	45	
**Age**,** years**	74 [63–82]	73 [62–81]	77 [65–84]	0.237
**Female sex**	166 (48.1)	145 (48.3)	21 (46.7)	0.835
**Hemisphere**	*N* = 340	*N* = 296	*N* = 44	0.579
Left	164 (47.5)	144 (48.6)	20 (45.5)	
Right	125 (36.2)	106 (35.8)	19 (43.2)	
Both	51 (14.8)	46 (15.5)	5 (11.4)	
**Territory**				
ICA/MCA/ACA	236 (68.4)	197 (65.7)	39 (86.7)	0.018
Vertebrobasilar	81 (23.5)	77 (25.7)	4 (8.9)	
Multiple	28 (8.1)	26 (8.7)	2 (4.4)	
**Baseline stroke severity**[12. 14]				< 0.001
Mild [NIHSS ≤ 8 points]	282 (81.7)	265 (88.3)	17 (37.8)	
Moderate [ NIHSS 9–15]	41 (11.9)	29 (9.7)	12 (26.7)	
Severe [ NIHSS ≥ 16]	22 (6.4)	6 (2.0)	16 (35.6)	
**Discharge NIHSS score** [12. 14]				< 0.001
Mild [≤ 8]	318 (92.2)	289 (96.3)	29 (64.4)	
Moderate [9–15]	21 (6.1)	8 (2.7)	13 (28.9)	
Severe [≥ 16]	6 (1.7)	3 (1.0)	3 (6.7)	
**Baseline mRS at presentation**				< 0.001
Good [0–2]	67 (19.4)	63 (21.0)	4 (8.9)	
Poor [3–5]	278 (80.6)	237 (79.0)	41 (91.1)	
**Discharge mRS**				< 0.001
Good [0–2]	206 (59.7)	193 (64.3)	13 (28.9)	
Poor [3–5]	136 (39.4)	106 (35.3)	30 (66.7)	
In hospital death [6]	3 (0.9)	1 (0.3)	2 (4.4)	
**Symptom onset to thrombolysis in min (range)**	*N* = 247	*N* = 222	*N* = 25	
	120 (90–165)	120 (90–168)	98 (69–142)	0.048
**CVRF**				
Arterial hypertension (*n* = 344)	254 (73.6)	226 (75.3)	28 (62.2)	0.121
Coronary heart disease (*n* = 343)	43 (12.5)	35 (11.7)	8 (17.8)	0.158
Heart failure (*n* = 344)	12 (3.5)	9 (3.0)	3 (6.7)	0.078
Atrial fibrillation (*n* = 345)	84 (24.3)	66 (22.0)	18 (40.0)	0.009
Diabetes mellitus (*n* = 345)	59 (17.1)	52 (17.3)	7 (15.6)	0.944
Smoking history (*n* = 343)	48 (13.9)	44 (14.7)	4 (8.9)	0.524
Hypercholesterolemia (*n* = 344)	69 (20.0)	60 (20.0)	9 (20.0)	0.771
Previous stroke (*n* = 344)	47 (13.7)	46 (15.3)	1 (2.2)	0.044
**Stroke etiology**				0.054
Arterio-arterial	91 (26.4)	81 (27.0)	10 (22.2)	
Cardio-embolic	136 (39.4)	111 (37.0)	25 (55.6)	
Small artery disease	38 (11.0)	38 (12.7)	0	
Vasculitis, dissection, coagulation disorder	13 (3.8)	11 (3.7)	2 (4.4)	
Unknown/more than one cause	67 (19.4)	59 (19.7)	8 (17.8)	
Duration of hospital stay (days)	8 (4–13)	8 (4–13)	8 (5–13)	0.718

ACA, A. cerebri anterior; CVRF, Cardiovascular risk factors; ICA, A. carotis internal; IQR, Interquartile Range; MCA, A. cerebri media; mRS, modified Rankin Scale; n, numbers; NIHSS, National Institutes of Health Stroke Scale. Data are presented as median [IQR] or n (%)

### Cardiovascular risk factors

**Fig. 2:**
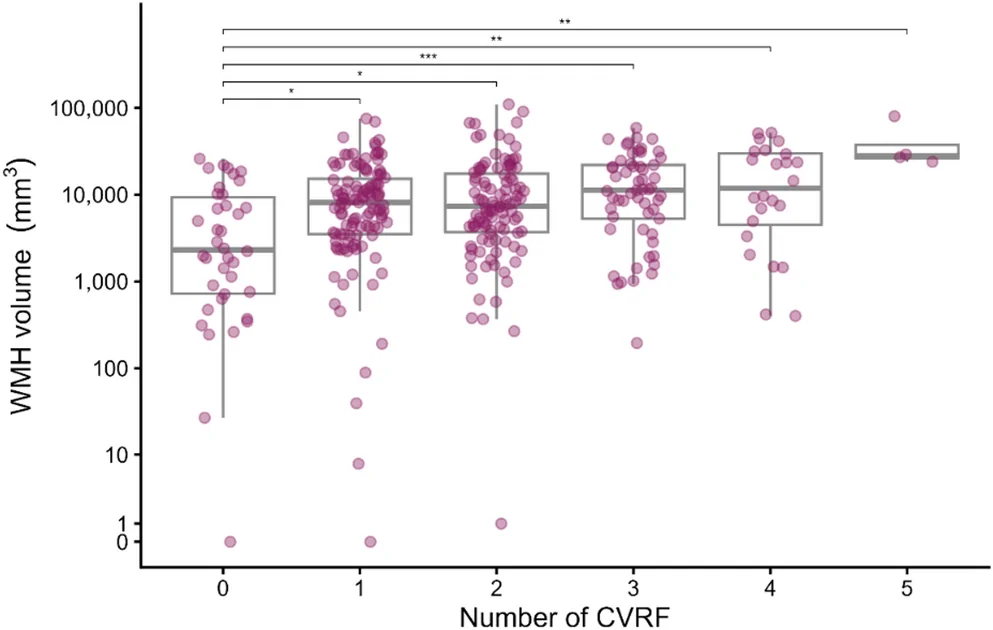
Cardiovascular risk factors (CVRF) and WMH at initial MRI The boxplot shows the WMH volume at the initial MRI for patients stratified by the number of CVRF they were affected by. A Kruskal-Wallis test revealed a significant difference in the initial WMH volume of patients between the CVRF groups (*p* < 0.001). Group differences were then evaluated by a post-hoc Dunn’s test with Bonferroni correction (*p < 0.05, **p < 0.01, ***p < 0.001). The boxes illustrate the median and IQR, with whiskers extending to ± 1.5 × IQR. Points represent individual patients. Abbreviations: CVRF, Cardiovascular Risk Factors; WMH, White Matter Hyperintensities.

Cardiovascular risk factors (CVRF) were common, with a majority of patients being affected by arterial hypertension (*n* = 254, 73.6%). The potential relationship between the number of CVRF and WMH volume at the initial MRI were therefore investigated (Fig. 2). When the patients were stratified based on the number of CVRF they were affected by, a Kruskal-Wallis test revealed a significant difference in the WMH volume between groups (*p* < 0.001). A post-hoc Dunn’s test with Bonferroni correction showed that patients with no CVRF had a significantly lower WMH volume at the initial MRI when compared to patients with one (*p* = 0.012), two (*p* = 0.013), three (*p* < 0.001), four (*p* = 0.003), or five (*p* = 0.002) CVRF. No significant difference in initial WMH was observed between the groups of patients with one to five CVRF. When Spearman’s rank correlation was applied, a significant but weak correlation was found between the number of CVRF and the initial WMH volume (ρ = 0.23, *p* < 0.001).

**Table 2:** Distribution of DWI lesions presented as medians with IQR

	1st MRI	2nd MRI
**Median total volume of DWI lesion in mm**^**3**^ [IQR]	1099.0 [216.6.6-3940.7] *n* = 345	1913.8 [420.3-8713.5] *n* = 345
**Volume of DWI lesion in patients with DWI reversal in mm**^**3**^ [IQR]	1638.5 [451.-5733.8] *n* = 76	460.2 [75.5-2130.6] *n* = 76
**Volume of DWI lesion in patients without DWI reversal in mm**^**3**^ [IQR]	1030.1 [172.3-3420.1] *n* = 269	2644.6 [809.4-11531.5] *n* = 269
	**DWI Reversal (≥ 25%)**, ***n**** = 76 (22.0%)*	
	**Absolute in mm**^**3**^ [IQR]	**Relative in %** [IQR]
**Median DWI-Reversal volume** [IQR]	879.9 [199.8-3443.7]	54.4 [IQR 43.8–81.9]

DWI, Diffusion-Weighted Imaging; IQR, Interquartile range

### DWI-reversal

**Fig. 3:**
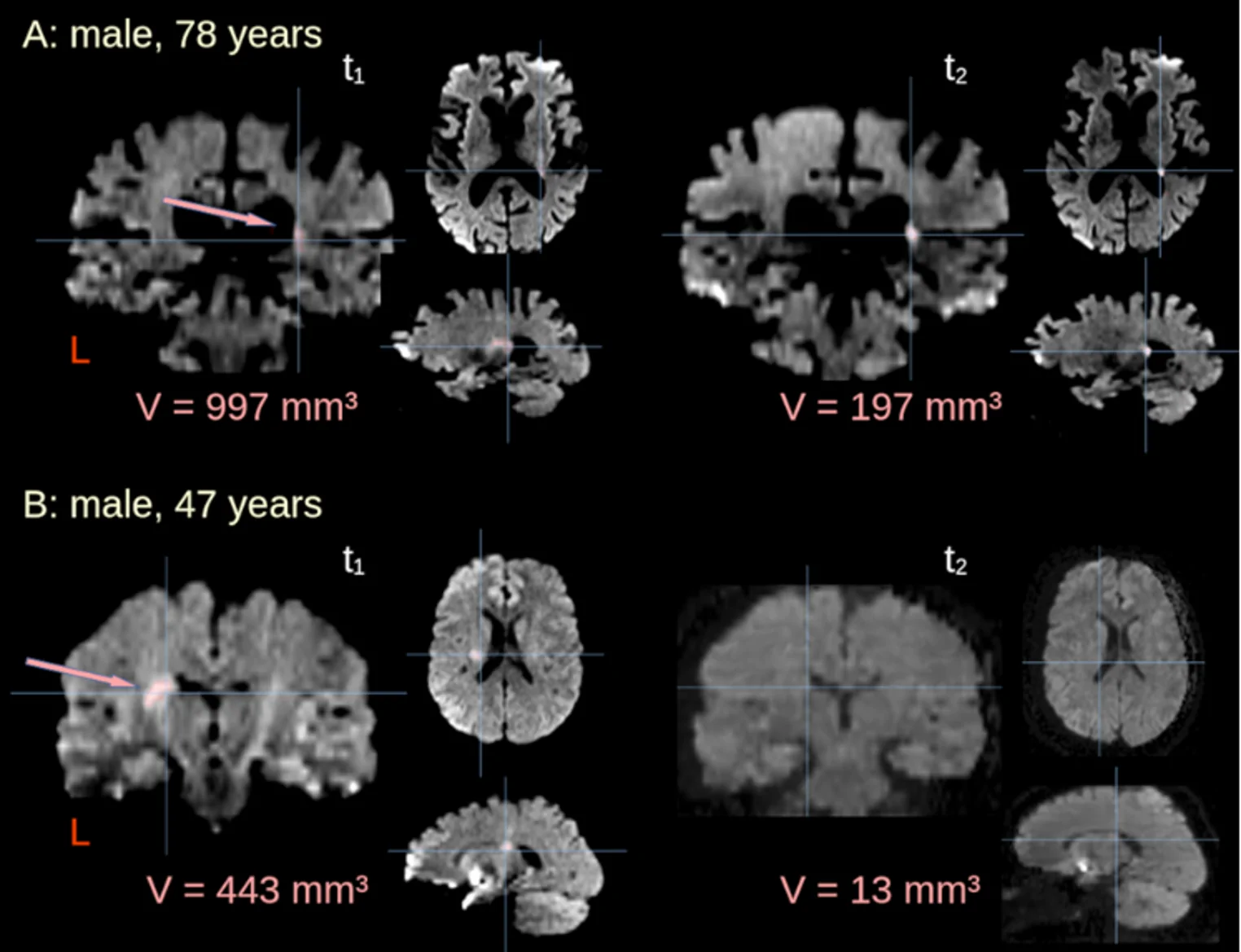
Examples of two patients with partial reversibility of DWI-hyperintense, ADC-hypointense acute ischemic stroke lesion. A: A patient (m, 78y) with ischemic stroke in the right MCA-territory, the DWI lesion volume of 997mm^3^ at timepoint t_1_ decreases significantly after thrombolysis to only 197mm^3^ at timepoint t_2_. B: A patient (m, 47y) with a DWI lesion in the left caudate nucleus. The initial DWI volume at timepoint t_1_ decreased by 97% at timepoint t_2_after thrombolysis

**Fig. 4:**
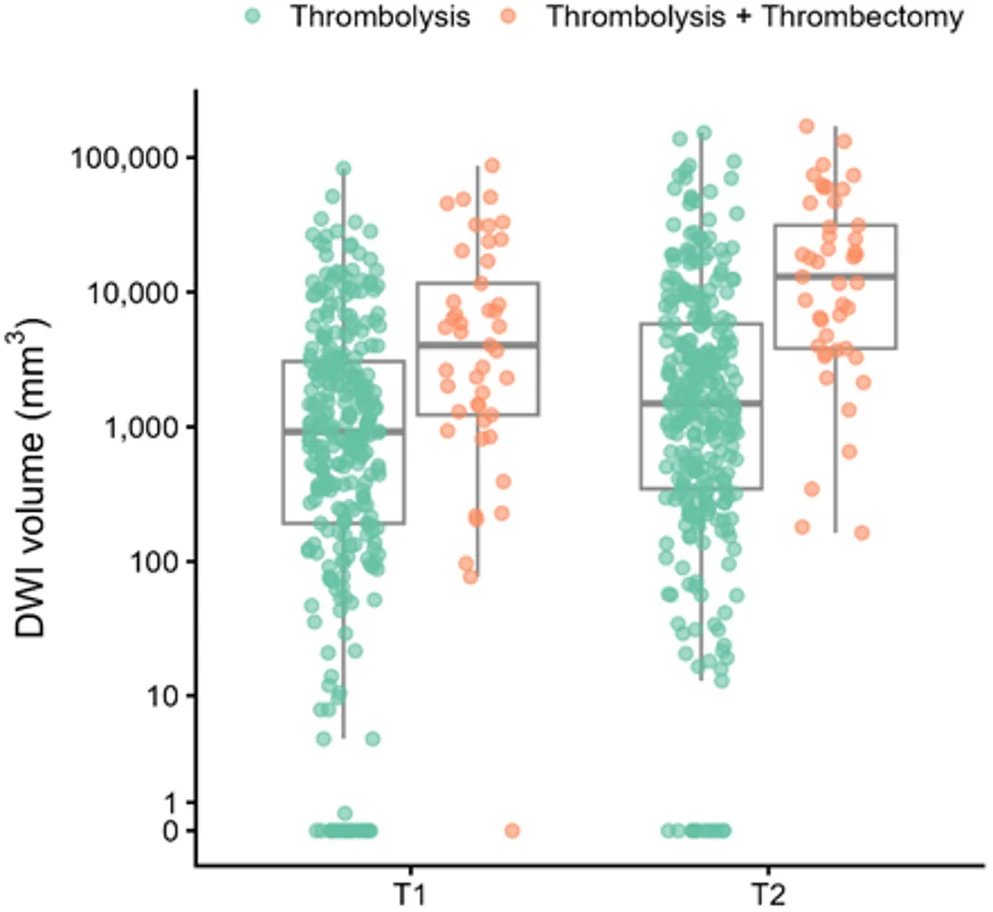
Course of DWI-lesion volumes before (T1) and after treatment (T2) with thrombolysis (green) or thrombolysis and thrombectomy (orange) on an individual patient level. A box plot comparing medians is used. Each dot represents a median value. Seven patients (2.0%) had DWI of 0 at both T1 and T2. The DWI lesion volumes were significantly higher in the thrombectomy group with a median lesion volume of 916.2 [IQR 190.9-3102.6] in the thrombolysis group before and 4033.1 [IQR 1168.7-14306.8] in the thrombectomy group before treatment (Wilcoxon rank sum test *p* < 0.001) and 1490.4 mm^3^ [IQR 345.0-5842.9] after treatment in the thrombolysis group and 13,014.5 mm^3^ [IQR 3756.1–38,643.5] in the thrombectomy group (*p* < 0.001)

DWI reversal, i.e. a decrease in infarct volume as identified as a decrease of DWI-lesion in brain MRI 2 of ≥ 25% of the initial DWI-hyperintense stroke lesion volume in brain MRI 1, was present in 76 patients (22.0%). The median DWI reversal volume was 879.9 mm^3^ as displayed in Table 2. The course of DWI-lesions on an individual and grouped patient level is displayed in Figs. 3 and 4. Supplementary Fig. 2 illustrates the individual changes of DWI volumes at T1 and T2 in a scatter plot.

Although the type of therapy in the univariate binary logistic regression model with DWI-reversal as main outcome variable showed a trend that DWI-reversal was less common when thrombolysis with additional thrombectomy was applied as compared to patients without additional thrombectomy (OR = 0.40, 95%CI 0.15–1.06, *p* = 0.066), it did not have a statistically significant influence on the occurrence of DWI reversal (Supplementary Table 1). Furthermore, in univariate binary logistic regression, the extent of initial infarct volume in DWI was not a significant predictor of DWI reversal (OR = 1.000005, 95% CI 0.99998-1.0000 *p* = 0.651). Markers of CSVD as WMH lesion volume in the baseline MRI were not associated with the probability of a DWI reversal (per mm³ increasing volume, OR 0.99999, 95% CI 0.9999–1.00004 *p* = 0.780), nor was the presence of CMBs (OR 0.73, 95% CI 0.29–1.84, *p* = 0.506). Similarly, the thrombolysis time window (on-label ≤ 4.5 h vs. imaging-based > 4.5 h after stroke onset) was not significantly associated with DWI reversal (OR 1.06, 95% CI 0.61–1.85, *p* = 0.830).

Because vascular territory differed between treatment groups, we performed additional sensitivity analyses. Among ICA/MCA/ACA strokes, DWI reversal occurred in 5/39 (12.8%) patients treated with thrombolysis plus thrombectomy and 49/197 (24.9%) treated with thrombolysis alone (OR 0.44, 95% CI 0.16–1.20). In vertebrobasilar strokes, the corresponding rates were 0/4 (0%) and 16/77 (20.8%), and in strokes involving multiple territories 0/2 (0%) and 6/26 (23.1%). The Mantel–Haenszel OR adjusted for vascular territory was 0.38 (95% CI 0.14–1.01), with no evidence of heterogeneity across strata (Breslow–Day *p* = 0.70). In a logistic regression model including vascular territory, the treatment estimate was similar (OR 0.38, 95% CI 0.14–1.01), while vascular territory itself was not associated with DWI reversal (vertebrobasilar OR 0.76, *p* = 0.39; multiple territories OR 0.85, *p* = 0.75).

With increasing NIHSS score (OR = 0.94, 95% CI 0.88-1.00, *p* = 0.044) and mRS score at admission (OR = 0.81, 95% CI 0.67–0.98, *p* = 0.030), the odds of a DWI reversal decreased.

In the multivariate logistic regression model as in the univariate model, patients with hypercholesterolemia were significantly more likely to experience DWI reversal (OR 2.91, 95% CI 1.60–5.28, *p* < 0.001, Bonferroni corrected *p* = 0.002, Table 3). The parameter of (history of) heart failure was not introduced into the multivariate model because of an n of only 12 cases. Coronary heart disease (OR 1.66, 95% CI 0.83–3.32, *p* = 0.155, Bonferroni corrected *p* = 0.775) and diabetes (OR 1.56, 95% CI 0.81–2.99, *p* = 0.186, Bonferroni corrected *p* = 0.93) were not associated with DWI-reversal. Other risk factors such as hypertension, atrial fibrillation, or the stroke etiology were not significantly associated with DWI-reversal.

DWI reversal rates did not differ across TOAST etiological categories: 25.3% in large-artery atherosclerosis, 18.4% in cardioembolism, 23.7% in small-vessel occlusion, 23.1% in other determined etiology, and 23.9% in undetermined etiology (Fisher’s exact test, *p* = 0.737). None of the individual etiological categories was significantly associated with DWI reversal in univariable logistic regression.

Pre-stroke statin use was not associated with DWI reversal in univariable analysis (OR 1.42, 95% CI 0.81–2.48, *p* = 0.22). When pre-stroke statin use was added to the multivariable model, the association between hypercholesterolemia and DWI reversal persisted (OR 3.14, 95% CI 1.69–5.85, *p* < 0.001), whereas statin use was not independently associated with DWI reversal (OR 0.86, *p* = 0.64).

**Table 3:** Multivariate logistic regression analysis with DWI-reversal (≥ 25%) as the main outcome variable (*n* = 76)

	OR	95% CI	*p*	Bonferroni Correction
Diabetes mellitus	1.56	0.81–2.99	0.186	0.93
Hypercholesterolemia	2.91	1.60–5.28	**< 0.001**	**0.002**
Coronary heart disease	1.66	0.83–3.32	0.155	0.775
NIHSS score at admission (per point increasing)	0.96	0.89–1.03	0.270	1.0
mRS admission (per point increasing)	0.83	0.65–1.05	0.120	0.6

A two-sided p-value  < 0.05 was considered statistically significant. DWI, Diffusion-weighted imaging; NIHSS, National Institutes of Health Stroke scale; mRS, modified Rankin scale

### White matter hyperintensities and cerebral microbleeds

The median WMH volume at the time of the baseline MRI scan was 8148.2 [IQR 3183.7–19,500.1] mm^3^ in the entire cohort. In the thrombolysis without thrombectomy cohort, the median was 8175.5 [IQR 3330.4–19,639.2] mm^3^, and in the thrombolysis with thrombectomy cohort, it was 8122.6 [IQR 2595.4–18,042.7] mm^3^. There was no significant difference between the treatment groups (*p* = 0.887) (Supplementary Tables 2 and Supplementary Fig. 1).

**Fig. 5:**
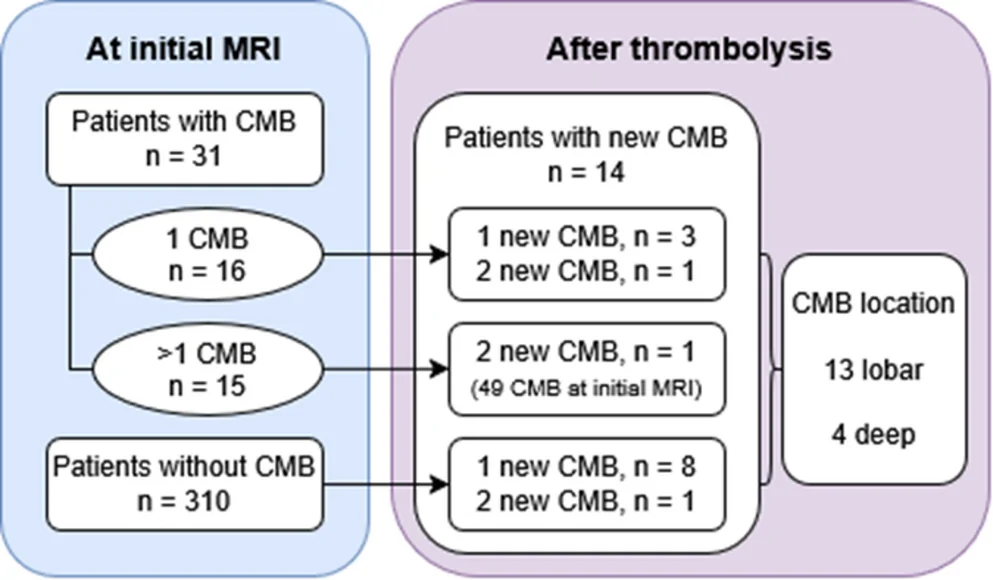
Presence of CMB at initial MRI and following thrombolysis. CMB were observed in 31/341 (9.1%) patients with available CMB data at the initial MRI, while a total of 14 (4.1%) patients developed new CMB following thrombolysis. CMB data were not available for four patients

Of the 31 patients with CMBs at baseline, 16 (52%) showed only one CMB before acute stroke treatment, whereas 15 had more than one CMB. Five of these patients (16%) developed at least one new CMB after thrombolysis. Among them, two patients developed two new CMBs, including one patient with a high baseline CMB burden of 49 CMBs. Of the 310 patients without CMBs at baseline, nine patients (2.9%) developed new CMBs after thrombolysis; eight developed one new CMB and one patient developed two new CMBs. Thus, a total of 17 new CMBs occurred in 14 patients. Of these 17 new CMBs, 13 (76%) were located in lobar regions and four (24%) in deep regions. Among the three patients who developed two new CMBs, one had one new deep and one new lobar CMB, whereas the other two patients each developed two new lobar CMBs. The baseline and newly detected CMB findings are summarized in Fig. 5.

To analyze the relationship between WMH volume and DWI reversal, the Mann-Whitney U test showed no significant difference (*p* = 0.502). Similarly, there was no significant association between WMH volume and the presence of CMBs (Mann-Whitney U test, *p* = 0.088) or between CMB presence and DWI reversal (chi-square test, *p* = 0.406). Thus, we found no evidence of an association between either baseline WMH volume or CMB presence and DWI reversal using the predefined ≥ 25% threshold.

In a sensitivity analysis using the more stringent threshold of ≥ 50% reduction in DWI lesion volume, 44/345 patients (12.8%) fulfilled the criterion for DWI reversal. The principal findings regarding WMH and CMBs remained unchanged: baseline WMH volume was not associated with ≥ 50% DWI reversal (*p* = 0.782), and ≥ 50% reversal occurred in 3/31 (9.7%) patients with CMBs compared with 41/314 (13.1%) patients without CMBs (Fisher’s exact test, *p* = 0.781).

### Early neurological improvement at 24 h

NIHSS at 24 h was available in 336 of 345 patients (97.4%). Patients with DWI reversal had lower NIHSS scores at 24 h than patients without DWI reversal (median 1 [IQR 0–3] vs. 2 [IQR 1–6], *p* = 0.004). ENI occurred in 44 of 73 patients (60.3%) with DWI reversal and in 130 of 263 patients (49.4%) without DWI reversal (*p* = 0.101). In multivariable logistic regression adjusting for baseline NIHSS and treatment modality, DWI reversal was not independently associated with ENI (OR 1.54, 95% CI 0.90–2.62, *p* = 0.112).

### Functional outcome

The median length of hospital stay was 8 days (IQR 4–13) in the entire cohort. Upon hospital discharge, 136 patients (39.4%) were classified with an mRS of 3 to 5. A further three patients (0.9%) died (mRS 6). An mRS of 3–6 was rated as poor functional outcome. In univariate logistic regression analysis with an mRS of 3–6, indicating an unfavorable clinical outcome at discharge (Supplementary Table 3), higher WMH volume was associated with a poorer functional outcome (per mm^3^ increasing OR 1.000024, 95% CI 1.000008-1.000039, *p* = 0.002). Furthermore, a larger DWI lesion volume at baseline was associated with a higher probability of worse functional outcome at discharge (per mm^3^ increasing DWI volume, OR 1.00003, 95% CI 1.000007-1.000057, *p* = 0.012), as shown in Supplementary Table 3. On the contrary, DWI reversal was associated with a lower risk of a poor functional outcome (OR 0.41, 95% CI 0.23–0.73, *p* = 0.003). In patients with CMBs, there was no association with functional outcome (Mann-Whitney U test, *p* = 0.572).

In the multivariable logistic regression model, only higher NIHSS values at baseline were independently associated with poor functional outcome (Table 4; OR 1.13, 95% CI 1.05–1.21, *p* = 0.001, Bonferroni-corrected *p* = 0.007). DWI reversal, WMH volume, baseline DWI lesion volume, treatment modality, and ICH were not independently associated with discharge outcome.

**Table 4:** Multivariate logistic regression model with a poor clinical outcome (mRS 3–6) as the main outcome variable

	OR	95% confidence interval		*p*	Bonferroni correction
		Lower value	Upper value		
**Age at admission**	1.01	0.99	1.03	0.313	1.0
**NIHSS score at admission (per point increasing)**	1.13	1.05	1.21	**0.001**	0.007
**Thrombolysis and thrombectomy vs. thrombolysis**	2.31	0.94	5.67	0.069	0.483
**DWI lesion volume at baseline**	1.00	1.00	1.00	0.861	1.0
**WMH volume 1st MRI**	1.00	1.00	1.00	0.450	1.0
**DWI reversal**	0.64	0.33	1.23	0.182	1.0
**ICH 2nd MRI (according to Heidelberg Bleeding Classification)**	1.15	0.37	3.54	0.807	1.0

DWI, Diffusion-Weighted Imaging; WMH, White Matter Hyperintensities; NIHSS, National Institutes of Health Stroke Scale; OR, Odds Ratio

### Intracerebral hemorrhage

The follow-up imaging showed an ICH in 21 patients (6.1%, c.f, Supplementary Table 4). These were mainly asymptomatic ICH as per definition, as five patients (1.4%) suffered a symptomatic ICH. Of those, sICH was fatal in two cases (Supplementary Table 5 for detailed description of intracranial hemorrhages). In univariate logistic regression analyses (Supplementary Table 6) with the complication of ICH after acute stroke treatment as main outcome variable, four factors were significantly associated with an increased likelihood for ICH development: (1) a higher NIHSS score at admission, (2) the treatment with additional thrombectomy, (3) a larger DWI hyperintense stroke lesion volume at the first MRI and (4) anticoagulation therapy at admission.

After adjustment for these covariates, combined treatment with thrombolysis and thrombectomy remained associated with ICH (OR 7.73, 95% CI 2.40–24.97, *p* < 0.001, corrected *p* = 0.004; Table 5). We found no evidence of an independent association of baseline CMB presence or WMH volume with ICH. Given the small number of hemorrhagic events, particularly only five sICH events, these negative findings should be interpreted cautiously.

**Table 5:** Multivariate logistic regression model with the occurrence of intracerebral hemorrhage (ICH) after stroke thrombolysis/thrombectomy as the main outcome variable

	OR	95% confidence interval		*p*	Bonferroni Correction
		Lower value	Upper value		
**Anticoagulation at admission**	3.81	0.84	17.40	0.084	0.336
**NIHSS score at admission (per point increasing)**	1.00	0.91	1.11	0.987	1.0
**DWI lesion volume at baseline**	0.999999	0.999950	1.000049	0.994	1.0
**Treatment with thrombolysis and thrombectomy (vs. thrombolysis alone)**	7.73	2.40	24.97	< 0.001	0.004

DWI, Diffusion-Weighted Imaging; NIHSS, National Institutes of Health Stroke Scale; OR, Odds Ratio

## Discussion

In this study, we investigated whether two chronic MRI markers of small-vessel brain injury, WMH and CMBs, were associated with early ischemic tissue evolution following reperfusion therapy in patients with acute ischemic stroke. DWI reversal occurred in approximately one fifth of patients, but we found no evidence of an association with WMH burden or CMB presence. Likewise, we found no evidence of an independent association of either marker with intracerebral hemorrhagic complications or discharge functional outcome. Although DWI reversal was associated with favorable functional outcome in univariable analysis, this association disappeared after adjustment for clinical covariates, whereas baseline stroke severity remained independently associated with outcome.

DWI reversal has been described as an imaging marker of early tissue recovery after successful reperfusion, although its reported frequency varies considerably depending on patient selection, treatment strategy, imaging intervals, and the definition of reversal. In our cohort, DWI reversal was observed in 22% of patients, which is within the range reported previously [22, 33]. While DWI lesions have traditionally been regarded as representing irreversible infarction, increasing evidence suggests that a proportion of diffusion abnormalities reflects potentially salvageable tissue if reperfusion is achieved sufficiently early [18, 21–23, 34]. Consequently, DWI reversal should not be interpreted as complete tissue recovery but rather as an imaging correlate of partial reversal of ischemic injury. The lack of an independent association between DWI reversal and functional outcome in our multivariable model further supports the concept that DWI reversal reflects favorable initial stroke characteristics rather than constituting an independent determinant of neurological recovery [34].

Our primary objective was to determine whether WMH burden and CMB presence modify early tissue reversibility after reperfusion therapy. From a pathophysiological perspective, an association is plausible because chronic small-vessel brain injury has been linked to endothelial dysfunction, impaired cerebrovascular regulation, and reduced cerebral blood flow [35–37]. However, we found no evidence of an association between WMH burden or CMB presence and DWI reversal in our cohort. These findings provide no evidence that these structural MRI markers are major determinants of early tissue reversibility; acute factors such as time to reperfusion, collateral circulation, and restoration of macrovascular blood flow may be more relevant. Conventional structural MRI markers may also incompletely reflect functional microcirculatory reserve during acute ischemia.

Although WMH and CMBs have previously been associated with outcome after ischemic stroke [14], their relationship with early infarct tissue reversibility remains incompletely characterized. Recent evidence further suggests that the prognostic relevance of WMH may depend not only on total lesion burden but also on their spatial distribution and associated structural network damage [38]. We hypothesized that greater chronic small-vessel brain injury might reduce the potential for DWI lesion reversal after reperfusion therapy. In this cohort, however, we found no evidence of an association between WMH burden or CMB presence and DWI reversal, including in the sensitivity analysis using the stricter ≥ 50% threshold.

The mechanism of the index stroke may itself influence lesion characteristics and tissue evolution. In the present cohort, however, DWI reversal rates did not differ detectably across TOAST etiological categories. This negative finding does not exclude a mechanistic effect of stroke etiology, particularly given the heterogeneity and sample sizes of the individual etiological groups.

An unexpected finding was the independent association between hypercholesterolemia and higher odds of DWI reversal. Because this was an exploratory analysis, the finding should be interpreted cautiously and should not be taken as evidence of a direct biological effect. We additionally considered pre-stroke statin therapy as a potential confounder. Adjustment for statin pretreatment did not materially alter the association between hypercholesterolemia and DWI reversal (OR 3.14, 95% CI 1.69–5.85), whereas statin pretreatment itself was not associated with DWI reversal (*p* = 0.64). Detailed information on statin type, dose, treatment duration, and other lipid-lowering therapies was not available; residual confounding therefore remains possible. This observation requires independent confirmation.

We further evaluated the occurrence of cerebral microbleeds after reperfusion therapy. Newly developed CMBs were observed in 4.1% of patients, consistent with previous reports from German stroke cohorts [39]. Patients with pre-existing CMBs more frequently developed additional microbleeds after treatment, and most newly detected lesions showed a lobar distribution. This is consistent with the observational study by Braemswig et al., who reported predominantly lobar new CMBs. Although such a pattern may be compatible with underlying cerebral amyloid angiopathy in some patients, possible or probable cerebral amyloid angiopathy was not documented as a diagnosis in our cohort.

Previous meta-analyses have reported an increased risk of intracerebral hemorrhage in patients with pre-existing CMBs undergoing intravenous thrombolysis [40–42], and a retrospective multicenter study similarly suggested increased post-thrombolytic hemorrhagic risk with higher CMB burden [43]. In our cohort, we found no evidence of an association between baseline CMB presence or WMH burden and hemorrhagic complications. However, only 31 patients had CMBs at baseline and only five sICH events occurred; therefore, these findings should not be interpreted as evidence that CMBs or WMH do not increase hemorrhagic risk. The limited event numbers substantially restrict statistical power.

Baseline stroke severity remained independently associated with discharge functional outcome, consistent with previous outcome studies [44–46]. The present findings should therefore be interpreted as showing limited additional prognostic information from WMH and CMBs within this cohort, rather than as evidence that chronic small-vessel pathology is irrelevant to recovery after ischemic stroke.

A major strength of this study is the standardized sequential MRI protocol, allowing assessment of temporal changes in ischemic lesion volume using comparable imaging time points. Furthermore, semi-automatic segmentation of both WMH and infarct volume enabled quantitative assessment with higher precision and lower interobserver variability than purely visual rating scales.

Several limitations should be acknowledged. First, this was a retrospective single-center study, limiting causal inference and generalizability. Second, inclusion required technically adequate baseline and follow-up MRI, which may have introduced selection bias. Although excluded cases could be identified, the clinical variables required for a reliable patient-level comparison were not systematically available in the analytical dataset and would have required retrospective linkage and, in part, additional review of the original clinical documentation. The direction and magnitude of this potential selection bias therefore could not be quantified. Third, pre-stroke mRS was not systematically documented, so pre-existing disability could not be incorporated into the outcome analyses. Fourth, variability in follow-up imaging timing may have influenced measured DWI reversal. Fifth, our definition was based on change in total lesion volume and did not use voxel-wise co-registration; it therefore cannot distinguish transient from sustained tissue-level reversal. Although the ≥ 50% sensitivity analysis yielded similar findings for WMH and CMBs, measurement variability remains particularly relevant for small lesions. Sixth, perfusion imaging was not routinely available. Seventh, functional outcome was assessed at hospital discharge rather than at 90 days. Finally, the relatively small numbers of patients undergoing thrombectomy, patients with baseline CMBs, and hemorrhagic events limited statistical power. Additional MRI features such as lacunes, enlarged perivascular spaces, and cortical superficial siderosis were beyond the scope of the present analysis; consequently, our findings should not be interpreted as an assessment of total CSVD burden.

In conclusion, DWI reversal occurred in approximately one fifth of patients after reperfusion therapy. We found no evidence that baseline WMH volume or CMB presence was associated with DWI reversal, and these findings were unchanged using a stricter ≥ 50% reversal threshold. We also found no evidence of an independent association of either marker with hemorrhagic complications or discharge functional outcome, although the low number of CMB-positive patients and hemorrhagic events limits inference. Future multicenter studies incorporating comprehensive small-vessel disease phenotyping, perfusion imaging, voxel-wise assessment of tissue fate, and long-term clinical follow-up are warranted.

## Conclusions

We found no evidence of an association between baseline white matter hyperintensity volume or cerebral microbleed presence and early DWI lesion reversal after reperfusion therapy in this cohort. These findings should be interpreted as specific to the MRI markers assessed rather than to overall cerebral small vessel disease burden.

## Supplementary Information

Below is the link to the electronic supplementary material.

Supplementary Material 1

Supplementary Material 2

Supplementary Material 3

Supplementary Material 4

Supplementary Material 5

Supplementary Material 6

Supplementary Material 7

Supplementary Material 8

## Data Availability

No datasets were generated or analysed during the current study.
