# Supplementary material for: Diffusion-weighted imaging reversal after reperfusion therapy in acute ischemic stroke: associations with white matter hyperintensities and cerebral microbleeds
Source: Neurol Res Pract. 2026 Oct 29;8(1):90. doi: 10.1186/s42466-026-00545-w (PMC13723929; doi:10.1186/s42466-026-00545-w)
Supplement: Supplementary file 1 [file 42466_2026_545_MOESM1_ESM.docx]

Supplementary Table 5: Intracranial hemorrhages according to Heidelberg Bleeding Classification

|  | ICHs according to Heidelberg Bleeding Classification | | Of those after Thrombectomy |
| --- | --- | --- | --- |
| Class | **Description** | **N (%)** | N (%) |
| 1 | **Hemorrhagic transformation of infarcted brain tissue** |  |  |
| 1a | HI1 | 5 (1.4) | 1 (20) |
| 1a/3c | HI1+3c | 1 (0.3) | 1 (100) |
| 1b | HI2 | 6 (1.7) | 1 (17)) |
| 1b/3c/3d | HI2, 3c, 3d | 1 (0.3) | 1 (100) |
| 1c | PH1 | 1 (0.3) | 1 (100) |
| 2 | **Intracerebral hemorrhage within and beyond infarcted brain tissue** |  |  |
| 2 | PH2 | 4 (1.2) | 2 (50) |
| 3 | 3c | 3 (0.9) | 3 (100) |
|  | **Total** | **21 (6.1)** | **10 (48)** |
|  | Of those sICH | 5 (1.4) | 2 (40) |

Abbreviations: sICH, symptomatic intracranial hemorrhage; HI1: Scattered small petechiae, no mass effect, HI2: Confluent petechiae, no mass effect, PH1: Hematoma within infarcted tissue, occupying <30%, no substantive mass effect, PH2: Hematoma occupying 30% or more of the infarcted tissue, with obvious mass effect, 3c: Subarachnoid hemorrhage, 3d: Subdural hemorrhage.
