# Supplementary material for: Diffusion-weighted imaging reversal after reperfusion therapy in acute ischemic stroke: associations with white matter hyperintensities and cerebral microbleeds
Source: Neurol Res Pract. 2026 Oct 29;8(1):90. doi: 10.1186/s42466-026-00545-w (PMC13723929; doi:10.1186/s42466-026-00545-w)
Supplement: Supplementary file 2 [file 42466_2026_545_MOESM2_ESM.docx]

Supplementary Table 6: Univariate logistic regression model with the occurrence of intracerebral hemorrhage (ICH) after stroke thrombolysis/thrombectomy as the main outcome variable

Abbreviations: CKD, chronic kidney disease; CMBs, cerebral microbleeds; DWI, Diffusion-Weighted Imaging; NIHSS, National Institute of Health Stroke Scale; OR, Odds Ratio; OTT, onset-to-treatment time; WMH, White Matter Hyperintensities

|  | **OR** | **95% confidence interval** | | **p** |
| --- | --- | --- | --- | --- |
|  |  | **Lower value** | **Upper value** |  |
| **Age at admission** | 1.01 | 0.97 | 1.04 | 0.749 |
| **Female Sex** | 0.80 | 0.33 | 1.95 | 0.619 |
| **NIHSS score at admission (per point increasing)** | 1.11 | 1.03 | 1.19 | **0.006** |
| **Treatment group** | 7.51 | 3.00 | 18.94 | **<0.001** |
| **DWI lesion volume at baseline** | 1.00 | 1.00 | 1.00 | **0.017** |
| **DWI reversal >25%** | 0.17 | 0.02 | 1.26 | 0.082 |
| **WMH volume 1^st^ MRI** | 1.00 | 1.00 | 1.00 | 0.502 |
| **Blood pressure adm. in mmHg** | 0.99 | 0.97 | 1.01 | 0.359 |
| **Hypercholesterolemia** | 0.19 | 0.03 | 1.42 | 0.105 |
| **Diabetes** | 1.47 | 0.53 | 4.06 | 0.459 |
| **Heart failure** | 1.17 | 0.17 | 8.03 | 0.875 |
| **Coronary heart disease** | 0.67 | 0.16 | 2.85 | 0.592 |
| **Atrial fibrillation** | 1.60 | 0.63 | 4.12 | 0.326 |
| **Arterial Hypertension** | 0.86 | 0.33 | 2.27 | 0.768 |
| **Thrombocytes (G/L)** | 1.00 | 1.00 | 1.01 | 0.871 |
| **Renal failure (CKD 3-5)** | 2.16 | 0.77 | 6.03 | 0.142 |
| **Anticoagulation at admission** | 5.31 | 1.34 | 21.11 | **0.018** |
| **CMBs in 1^st^ MRI** | 1.00 | 0.22 | 4.47 | 0.995 |
| **OTT>4.5 h** | 0.55 | 0.18 | 1.68 | 0.294 |
| **Cardioembolic stroke** | 1.43 | 0.59 | 3.46 | 0.430 |
| **Arterio-arterial stroke** | 0.45 | 0.13 | 1.56 | 0.205 |
