# Supplementary material for: Diffusion-weighted imaging reversal after reperfusion therapy in acute ischemic stroke: associations with white matter hyperintensities and cerebral microbleeds
Source: Neurol Res Pract. 2026 Oct 29;8(1):90. doi: 10.1186/s42466-026-00545-w (PMC13723929; doi:10.1186/s42466-026-00545-w)
Supplement: Supplementary file 3 [file 42466_2026_545_MOESM3_ESM.docx]

* n=7 for DWI volume of 0 mm^3^ at T1 and T2

**Supplementary Figure 2:** Evolution of DWI lesion volumes with individuals values before (T1) and after acute stroke therapy (T2). Patients treated with thrombolysis and thrombectomy show a tendency to larger DWI volumes (orange dots). Axes have a pseudo-logarithmic (base 10) transformation.
