# Supplementary material for: Diffusion-weighted imaging reversal after reperfusion therapy in acute ischemic stroke: associations with white matter hyperintensities and cerebral microbleeds
Source: Neurol Res Pract. 2026 Oct 29;8(1):90. doi: 10.1186/s42466-026-00545-w (PMC13723929; doi:10.1186/s42466-026-00545-w)
Supplement: Supplementary file 4 [file 42466_2026_545_MOESM4_ESM.docx]

**Supplementary Table 2:** Analysis of CSVD markers among the treatment groups before treatment

|  | **Total** | **Thrombolysis** | **Thrombolysis + thrombectomy** | **p-value** |
| --- | --- | --- | --- | --- |
| **n** | 345 | 300 | 45 |  |
| **WMH-load (mm^3^), median [IQR]** | 8148.2 [3183.7-19,500.1] | 8175.5 [3330.4-19,639.2] | 8122.6 [2595.4-18,042.7] | 0.887* |
| **WMH-load (mm^3^), min-max** | 0.0-109,638.6 | 0.0-109,638.6 | 266.9-43,884.4 |  |
| **CMB, n (%), n=341** | 31 (9.1) | 28 (9.3) | 3 (6.7) | 0.539# |
| *CMB undeterminable, n (%)* | *4 (1.2)* | *4 (1.3)* | *0* |  |
| *Wilcoxon rank sum test, #Chi square test | | | | |
