# Supplementary material for: Diffusion-weighted imaging reversal after reperfusion therapy in acute ischemic stroke: associations with white matter hyperintensities and cerebral microbleeds
Source: Neurol Res Pract. 2026 Oct 29;8(1):90. doi: 10.1186/s42466-026-00545-w (PMC13723929; doi:10.1186/s42466-026-00545-w)
Supplement: Supplementary file 5 [file 42466_2026_545_MOESM5_ESM.docx]

Supplementary Table 3: Univariate logistic regression model with a poor clinical outcome as the outcome variable (mRS 3-6)

Abbreviations: DWI, Diffusion-Weighted Imaging; mRS, modified Ranking Scale; NIHSS, National Institute of Health Stroke Scale; OR, Odds Ratio; CMBs: Cerebral Microbleeds; WMH, White Matter Hyperintensities; CKD Chronic kidney Disease; ICH Intracranial hemorrhage; MRI, Magnetic Resonance Imaging; OTT, Onset-to-treatment-time

|  | **OR** | **95% confidence interval** | | **p** |
| --- | --- | --- | --- | --- |
|  |  | **Lower value** | **Upper value** |  |
| **Age at admission** | 1.02 | 1.01 | 1.04 | 0.006 |
| **Female Sex** | 1.28 | 0.83 | 1.97 | 0.261 |
| **NIHSS score at admission (per point increasing)** | 1.15 | 1.10 | 1.22 | <0.001 |
| **Treatment group thrombolysis and thromectomy** | 4.44 | 2.24 | 8.82 | <0.001 |
| **DWI lesion volume at baseline** | 1.000032 | 1.000007 | 1.000057 | 0.012 |
| **WMH volume 1^st^ MRI** | 1.000024 | 1.000024 | 1.000039 | 0.002 |
| **CMBs 1^st^ MRI** | 1.44 | 0.69 | 3.01 | 0.337 |
| **Hypercholesterolemia** | 0.93 | 0.60 | 1.75 | 0.930 |
| **Diabetes** | 1.53 | 0.89 | 2.64 | 0.128 |
| **Heart failure** | 1.86 | 0.67 | 5.18 | 0.232 |
| **Coronary heart disease** | 0.98 | 0.55 | 1.83 | 0.984 |
| **Arterial Hypertension** | 1.54 | 0.93 | 2.53 | 0.091 |
| **Renal failure (CKD 3-5)** | 1.84 | 1.02 | 3.31 | 0.043 |
| **ICH 2^nd^ MRI (according to Heidelberg Bleeding Classification)** | 2.55 | 1.03 | 6.34 | 0.043 |
| **DWI reversal** | 0.41 | 0.23 | 0.73 | 0.003 |
| **Hospital stay in days** | 1.02 | 0.99 | 1.05 | 0.137 |
| **OTT>4.5 h** | 0.85 | 0.53 | 1.38 | 0.516 |
