# Supplementary material for: Diffusion-weighted imaging reversal after reperfusion therapy in acute ischemic stroke: associations with white matter hyperintensities and cerebral microbleeds
Source: Neurol Res Pract. 2026 Oct 29;8(1):90. doi: 10.1186/s42466-026-00545-w (PMC13723929; doi:10.1186/s42466-026-00545-w)
Supplement: Supplementary file 6 [file 42466_2026_545_MOESM6_ESM.docx]

Supplementary Table 4: Intracerebral hemorrhages according to Heidelberg Bleeding Classification.

|  | ICHs according to Heidelberg Bleeding Classification | | |
| --- | --- | --- | --- |
| Class | Description | n | % |
| 1a | HI1 | 5 | 1.4 |
| 1a/3c | HI1+3c | 1 | 0.3 |
| 1b | HI2 | 6 | 1.7 |
| 1b/3c/3d | HI2, 3c, 3d | 1 | 0.3 |
| 3c | 3c | 3 | 0.9 |
| 1c | PH1 | 1 | 0.3 |
| 2 | PH2 | 4 | 1.2 |
|  | **Total** | **21** | **6.1** |
|  | Of those sICH | 5 | 1.4 |

Abbreviations: sICH, symptomatic intracranial hemorrhage; HI1: Scattered small petechiae, no mass effect, HI2: Confluent petechiae, no mass effect, PH1: Hematoma within infarcted tissue, occupying <30%, no substantive mass effect, PH2: Hematoma occupying 30% or more of the infarcted tissue, with obvious mass effect, 3c: Subarachnoid hemorrhage, 3d: Subdural hemorrhage.
