# Supplementary material for: Diffusion-weighted imaging reversal after reperfusion therapy in acute ischemic stroke: associations with white matter hyperintensities and cerebral microbleeds
Source: Neurol Res Pract. 2026 Oct 29;8(1):90. doi: 10.1186/s42466-026-00545-w (PMC13723929; doi:10.1186/s42466-026-00545-w)
Supplement: Supplementary file 7 [file 42466_2026_545_MOESM7_ESM.docx]

**Supplementary Table 1: Univariate logistic regression model with DWI-reversal (> 25%) as the main outcome variable (n=72)**

|  | **OR** | **95% confidence interval** | **p** |
| --- | --- | --- | --- |
| Age | 1.00 | 0.981-1.018 | 0.934 |
| Hypertension (n=344) | 1.38 | 0.76-2.52 | 0.293 |
| Diabetes mellitus (n=344) | 1.93 | 1.06-3.50 | **0.031** |
| History of smoking (n=343) | 1.81 | 0.98-3.36 | 0.058 |
| Hypercholesterolemia (n=344) | 3.04 | 1.71-5.40 | **<0.001** |
| Coronary heart disease (n=343) | 2.12 | 1.13-3.96 | **0.019** |
| Heart failure (n=344) | 3.22 | 1.14-9.02 | **0.027** |
| Atrial fibrillation (n=345) | 0.79 | 0.43-1.46 | 0.449 |
| Treatment group (thrombolysis and thrombectomy) | 0.40 | 0.15-1.06 | 0.066 |
| DWI lesion volume at baseline | 1.00 | 1.00-1.00 | 0.651 |
| WMH volume at baseline | 1.00 | 1.00-1.00 | 0.780 |
| CMB in 1^st^ MRI (n=343) | 0.732 | 0.29-1.84 | 0.506 |
| OTT >4.5 h or unknown (vs. <4.5h) (n=247) | 1.06 | 0.61-1.85 | 0.830 |
| OTT >3h or unknown (vs. <3h) (n=247) | 1.04 | 0.62-1.73 | 0.895 |
| ICH in 2^nd^ MRI | 0.167 | 0.02-1.26 | 0.082 |
| NIHSS score at admission (per point increasing) | 0.94 | 0.88-1.00 | **0.044** |
| mRS admission (0-5) | 0.81 | 0.67-0.98 | **0.030** |
| Large-artery atherosclerosis | 1.3 | 0.73-2.25 | 0.385 |
| Cardioembolism | 0.70 | 0.41-1.19 | 0.189 |
| Small-vessel occlusion | 1.12 | 0.50-2.46 | 0.794 |
| Other determined etiology | 1.06 | 0.29-3.97 | 0.926 |
| Undetermined etiology | 1.14 | 0.61-2.14 | 0.684 |
| Posterior vs. Anterior Circulation stroke (n=327) | 0.72 | 0.39-1.32 | 0.285 |
