# Supplementary material for: Diffusion-weighted imaging reversal after reperfusion therapy in acute ischemic stroke: associations with white matter hyperintensities and cerebral microbleeds
Source: Neurol Res Pract. 2026 Oct 29;8(1):90. doi: 10.1186/s42466-026-00545-w (PMC13723929; doi:10.1186/s42466-026-00545-w)
Supplement: Supplementary file 8 [file 42466_2026_545_MOESM8_ESM.docx]

**Supplementary Figure 1:** Distribution of WMH among the treatment groups. There was no significant difference between the treatment groups (Wilcoxon rank sum test, p=0.887.
